# Further Evidence that Inhibition of Neuronal Voltage-Gated Calcium Channels Contributes to the Hypnotic Effect of Neurosteroid Analogue, 3β-OH

**DOI:** 10.3389/fphar.2022.850658

**Published:** 2022-05-23

**Authors:** Tamara Timic Stamenic, Francesca M. Manzella, Stefan Maksimovic, Kathiresan Krishnan, Douglas F. Covey, Vesna Jevtovic-Todorovic, Slobodan M. Todorovic

**Affiliations:** ^1^ Department of Anesthesiology, University of Colorado, Anschutz Medical Campus, Aurora, United States; ^2^ Neuroscience Graduate Program, University of Colorado, Anschutz Medical Campus, Aurora, United States; ^3^ Department of Developmental Biology, Washington University School of Medicine, Saint Louis, MO, United States; ^4^ Taylor Family Institute for Innovative Psychiatric Research, Washington University School of Medicine, Saint Louis, MO, United States

**Keywords:** voltage-gated calcium channels, R-type calcium channels, hypnosis, EEG, neuroactive steroids, thalamus, *ex vivo* in slice electrophysiology

## Abstract

We recently reported that a neurosteroid analogue with T-channel-blocking properties (3β,5β,17β)-3-hydroxyandrostane-17-carbonitrile (3β-OH), induced hypnosis in rat pups without triggering neuronal apoptosis. Furthermore, we found that the inhibition of the Ca_V_3.1 isoform of T-channels contributes to the hypnotic properties of 3β-OH in adult mice. However, the specific mechanisms underlying the role of other subtypes of voltage-gated calcium channels in thalamocortical excitability and oscillations *in vivo* during 3β-OH-induced hypnosis are largely unknown. Here, we used patch-clamp recordings from acute brain slices, *in vivo* electroencephalogram (EEG) recordings, and mouse genetics with wild-type (WT) and Ca_V_2.3 knock-out (KO) mice to further investigate the molecular mechanisms of neurosteroid-induced hypnosis. Our voltage-clamp recordings showed that 3β-OH inhibited recombinant Ca_V_2.3 currents. In subsequent current-clamp recordings in thalamic slices *ex vivo*, we found that selective Ca_V_2.3 channel blocker (SNX-482) inhibited stimulated tonic firing and increased the threshold for rebound burst firing in WT animals. Additionally, in thalamic slices we found that 3β-OH inhibited spike-firing more profoundly in WT than in mutant mice. Furthermore, 3β-OH reduced bursting frequencies in WT but not mutant animals. In ensuing *in vivo* experiments, we found that intra-peritoneal injections of 3β-OH were less effective in inducing LORR in the mutant mice than in the WT mice, with expected sex differences. Furthermore, the reduction in total α, β, and low γ EEG power was more profound in WT than in Ca_V_2.3 KO females over time, while at 60 min after injections of 3β-OH, the increase in relative β power was higher in mutant females. In addition, 3β-OH depressed EEG power more strongly in the male WT than in the mutant mice and significantly increased the relative δ power oscillations in WT male mice in comparison to the mutant male animals. Our results demonstrate for the first time the importance of the Ca_V_2.3 subtype of voltage-gated calcium channels in thalamocortical excitability and the oscillations that underlie neurosteroid-induced hypnosis.

## Introduction

The mechanisms whereby general anesthetics produce a loss of consciousness are not well understood, but it is well known that most general anesthetics currently in use have either N-methyl-d-aspartate (NMDA) receptor-blocking and/or γ-aminobutyric acid A (GABA_A_) receptor-enhancing properties. The idea that neuroactive steroids have sedative/hypnotic properties has been around since the introduction of alphaxalone ((3α,5α)3-hydroxypregnane-11,20-dione) (Lau et al., 2013; Arenillas and Gomez de Segura, 2018). Many neuroactive steroids act as positive modulators of GABA_A_ receptors and can be effective modulators of other receptors (serotonin, NMDA, α2-adrenergic) or voltage-gated calcium (L- and T-type) ion channels (Lambert et al., 1995; Rupprecht, 2003; Stell et al., 2003; Belelli and Lambert, 2005; Tuem and Atey, 2017).

Since most used anesthetics produce unwanted effects in the pediatric population, the quest for novel, safer anesthetics is one of the most important and urgent tasks in the anesthesiology field. Every year, more than 4 million children are exposed to general anesthetics due to diagnostic or surgical procedures. Since it has been shown that common general anesthetics could be harmful to brain development, triggering neurodegeneration and cognitive deficits, the US Food and Drug Administration (FDA) issued a warning about the potentially neurotoxic effects of general anesthetics in children (https:// www.fda.gov/drugs/drug-safety-and-availability/fda-drug-safety-communication-fda-review-results-new-warnings-about-using-general-anesthetics-and#:∼:text=%5B%2012%2D14%2D2016%20%5D,the%20development%20of%20children’s%20brains). We previously reported that the neurosteroid analogue (3β,5β,17β)-3-hydroxyandrostane-17-carbonitrile (3β-OH) induces hypnosis in rat pups without triggering neuronal apoptosis and acts as a thalamic Ca_V_3.1 T-type calcium channel (T-channel) blocker without having an effect on the synaptic and extra-synaptic γ-aminobutyric acid A (GABA_A_) receptors (Atluri et al., 2018; Timic Stamenic et al., 2021). We also reported that 3β-OH blocks low-voltage-gated calcium T-channels in the thalamic reticular nucleus (TRN), the central medial nucleus of the thalamus (CMT), and the sensory neurons of the dorsal root ganglion (DRG) (Todorovic et al., 2004; Joksovic et al., 2007; Timic Stamenic et al., 2021), but its effect on other voltage-gated calcium channels remains unknown. Since it is well known that most hypnotics and general anesthetics interact with multiple targets, we sought to determine the additional mechanisms underlying 3β-OH-induced hypnosis in mice.

The Ca_V_2.3 isoform of voltage-gated R-type calcium channels (R-type channels) (Soong et al., 1993) is implicated in both presynaptic neurotransmitter release and postsynaptic somatodendritic integration and long-term potentiation (Breustedt et al., 2003; Dietrich et al., 2003; Kamp et al., 2005; Catterall, 2011). Similar to other high-voltage-activated (HVA) calcium channels, R-type channels not only contribute to neurotransmitter release (Wu et al., 1998; Ricoy and Frerking, 2014) but are involved in the regulation of neuronal excitability (Metz et al., 2005; Zaman et al., 2011; Zamponi, 2016). Most of the Ca_V_2.3 channels are encoded by the *cacna1e* gene and expressed in different splice variants in the brain (Williams et al., 1994; Schneider et al., 2020). It is well known that R-type channels are modulated by protein phosphorylation (Neumaier et al., 2018; Schneider et al., 2018), glutamate and trace metals such as Zn^2+^ and Cu^2+^ (Shcheglovitov et al., 2012; Neumaier et al., 2020), and G protein-coupled receptors (GPCRs) (Berecki et al., 2016; Jeong et al., 2016). The R-type channel is also known to play a role in epileptogenesis in rodents, and its deletion reduces susceptibility to chemically induced seizures (Weiergräber et al., 2006, 2007; Zaman et al., 2011; Simms and Zamponi, 2014; Helbig et al., 2018). Changes in the expression of Ca_V_2.3 channels were also reported in Parkinson’s disease (Benkert et al., 2019; Schneider et al., 2020) and pain processing (Saegusa et al., 2000, 2002). Importantly, we showed that Ca_V_2.3 channels are important molecular target for the effects of the volatile anesthetic isoflurane in the TRN (Joksovic et al., 2009). Additionally, Ca_V_2.3-deficient mice exhibited reduced wake duration (spontaneous or urethane-induced), increased slow-wave sleep (SWS), changes in sleep stage transitions, and altered electroencephalographic (EEG) amplitudes (Siwek et al., 2014). Previous molecular studies have demonstrated that Ca_V_2.3 calcium channels are abundantly expressed in the brain, with relatively low expression in thalamocortical projection neurons (Williams et al., 1994; Parajuli et al., 2012) and a higher expression in GABAergic interneurons of the TRN (Weiergräber et al., 2008). However, since the inhibitory TRN projects to the majority of thalamic nuclei (Scheibel and Scheibel, 1966; Steriade et al., 1984; Kolmac and Mitrofanis, 1997), it is reasonable to infer that the global deletion of R-type channels may alter excitability across the whole thalamus. The central medial nucleus of thalamus (CMT) is a part of the intralaminar thalamus with diffuse projections to the anterior and posterior regions of the cortex, the nucleus accumbens, claustrum, the caudate-putamen, the olfactory tubercle, and the amygdala (Vertes et al., 2012). It has been proposed that CMT acts as a key hub through which general anesthesia and natural sleep are initiated (Baker et al., 2014).

Here, we used *in vitro* (recordings from HEK cells) and *ex vivo* (CMT slices recordings) molecular studies with qPCR and *in vivo* EEG electrophysiology with behavioral testing to investigate the possible role of Ca_V_2.3 R-channels in the hypnotic effect of 3β-OH in mice. We already demonstrated dose-dependent sex differences in 3β-OH-induced hypnosis and EEG changes in rats (Joksimovic et al., 2021), and consequently investigated sex-dependent neurosteroid effects using mouse genetics in this study.

## Materials and Methods

### Animals

Experimental procedures with animals were performed according to the guidelines approved by the University of Colorado Anschutz Medical Campus. The treatments of animals adhered to the guidelines set out in the NIH *Guide for the Care and Use of Laboratory Animals*. All efforts were made to minimize animal suffering and to use only the number of animals necessary to produce reliable scientific data. Male juvenile and adult (postnatal day (P), P35-50) wild-type (WT) and Ca_V_2.3 knock-out (KO) mice were used for *ex vivo* electrophysiological recordings, while older male and female WT and Ca_V_2.3 KO mice (3 months) were used for behavioral and *in vivo* electrophysiological experiments. The generation of the Cav2.3 null mutant (*cacna1e*, α1E null) and WT littermates has previously been described in detail (Wilson et al., 2000; Pereverzev et al., 2002; Weiergräber et al., 2006) and used for all experiments. All animals were maintained on a 14/10 h light–dark cycle with food and water ad libitum.

### Loss of Righting Reflex

LORR is commonly used to assess the depth of hypnosis in animal models. LORR is assessed by placing the mouse on its back until the animal loses its righting reflex. The criterion for the LORR is the mouse’s failure to right itself twice within a 30 s period. In addition, the mouse is considered to have regained its righting reflex when it can right itself within a 30 s period. In order to test the effect of 3β-OH on LORR, male and female WT and Ca_V_2.3 KO mice were injected intraperitoneally (i.p.) with 80 mg/kg of 3β-OH and LORR duration was analyzed. We chose this dose based on our previous study, which showed that about 50% of WT mice exhibited LORR when injected with 80 mg/kg of 3β-OH i.p. (Timic Stamenic et al., 2021).

### Ribonucleic Acid Extraction and Quantitative Polymerase Chain Reaction

Animals were euthanized with isoflurane and their tissue (whole thalamus or CMT punches) was collected. In order to obtain a higher yield of RNA, punched samples of CMT were pooled from three animals per group. RNA was extracted using the RNeasy mini kit (Qiagen) according to the manufacturer’s protocol. RNA purity was checked using the NanoDrop Spectrophotometer, OneC (Thermo Fisher Scientific). For each sample, 50 ng of pure RNA was used to synthesize cDNA based on the instructions for the iScript Advance cDNA kit (BioRad) over four cycles: 25°C for 5 min, 46°C for 30 min, 95°C for 5 min, and 4°C for 30 min qPCR was performed for all samples using TaqMan gene expression assay (probes: *cacna1e*- Mm00494444_m1; *gapdh*- Mm99999915_g1; all probes were obtained from Thermo Fisher Scientific). The level of expression of *cacna1e* was normalized to the housekeeping gene (*gapdh*) based on formula 2^(-ΔCq) for all samples.

### HEK-293 Cells

For recordings of Ca_V_2.3 high-voltage-activated (HVA) calcium current in HEK-293 recombinant cells, we used internal solution containing 110 mM Cs-methane sulfonate, 14 mM phosphocreatine, 10 mM HEPES, 9 mM EGTA, 5 mM MgATP, and 0.3 mM Tris-GTP, with the pH adjusted to 7.15–7.20 with CsOH. The external solution used to record recombinant Ca_V_2.3 calcium currents contained 2 mM BaCl_2_, 152 mM TEA-Cl, and 10 mM HEPES adjusted to pH 7.4 with TEA-OH. In most experiments, during recordings of recombinant HVA currents, a P/5 protocol was used for on-line leakage subtractions. HEK-293 cells were stably transfected with both the α1E (Ca_V_2.3) and β3 calcium channel subunits, as described previously (Nakashima et al., 1998). Cells were typically used 1–4 d after plating. The steps used to activate Ca_V_2.3-generated currents in HEK-293 cells in whole-cell experiments were typically based on a holding potential (Vh) of −70 mV to test potentials (Vt) at 0 mV.

### *Ex Vivo* Brain Slice Preparation

The WT and Ca_V_2.3 KO mice were briefly anesthetized with 5% isoflurane and decapitated. Their brains were removed rapidly and placed in a cold (4°C) oxygenated (95 vol% O_2_ and 5 vol% CO_2_) solution. Live 250 μm-thick coronal brain slices were sectioned at 4°C using a vibrating micro slicer in the same cold solution (in mM): sucrose 260, d-glucose 10, NaHCO_3_ 26, NaH_2_PO_4_ 1.25, KCl 3, CaCl_2_ 2, MgCl_2_ 2(Laica VT 1200S). Brain slices were immediately incubated for 30 min in the following solution (in mM): NaCl 124, d-glucose 10, NaHCO_3_ 26, NaH_2_PO_4_ 1.25, KCl 4, CaCl_2_ 2, MgCl_2_ 2. This was carried out at 37 °C before use in electrophysiology experiments, which were carried out at room temperature. During incubation, the slices were constantly perfused with a gas mixture of 95 vol% O_2_ and 5 vol% CO_2_.

### Electrophysiology Experiments in Brain Slices

The external solution for current-clamp electrophysiology experiments consisted of the following (in mM): NaCl 125, d-glucose 25, NaHCO_3_ 25, NaH_2_PO_4_ 1.25, KCl 2.5, MgCl_2_ 1, CaCl_2_ 2. For current-clamp experiments, the external solution contained the synaptic blockers picrotoxin (20 μM), D-2-amino-5-phosphonovalerate (D-AP5; 50 µM), and 2,3-dihydroxy-6-nitro-7-sulfamoyl-benzo[f]quinoxaline-2,3-dione (NBQX; 5 µM). The internal solution used for the current-clamp recordings consisted of the following (in mM): potassium-d-gluconate 130, ethylene-glycol-bis(β-aminoethylether)N,N,N′,N′-tetraacetic acid (EGTA) 5, NaCl 4, CaCl_2_ 0.5, HEPES 10, Mg ATP 2, Tris GTP 0.5, pH 7.2.

Whole-cell recordings were performed in CMT neurons visualized using Zeiss optics (Zeiss AXIO Examiner D1, ×40 objective). Glass microelectrodes (Sutter Instruments, borosilicate glass with filament OD 1.2 mm) were pulled using a Sutter Instruments P-1000 model and fabricated to maintain an initial resistance of 3–6 mΩ. Neuronal membrane responses were recorded using a Multiclamp 700 B amplifier (Molecular Devices, Foster City, CA, United States). Voltage current commands and the digitization of the resulting voltages and currents were performed with the Clampex 8.3 software (Molecular Devices) running on a PC-compatible computer. Resulting current traces were analyzed using Clampfit 10.5 (Molecular Devices). Statistical and graphical analyses were performed using the GraphPad Prism 9.0 software (GraphPad Software) or Origin 7.0 (OriginLab). Results are typically presented as means ± SEM unless stated otherwise.

### Current-Clamp Experiments

Both stimulated tonic and burst firing properties of CMT neurons were characterized using multistep protocols in WT and Ca_V_2.3 KO mice. To investigate the stimulated tonic firing patterns in CMT cells, we injected a depolarizing current pulse through the recording pipette with a 400 ms duration in 25 pA incremental steps starting from 50 pA. To investigate rebound burst firing patterns, the neurons were injected with hyperpolarizing currents in 25 pA intervals from 0 to −225 pA. Subsequent stimulated tonic action potential (AP) frequencies, rebound burst firing thresholds, AP in rebound burst, and input resistances (IR) were determined. The resting membrane potential (RMP) was measured at the beginning of each recording and not corrected for the liquid junction potential.

### Electroencephalogram and Local Field Potential Data Acquisition and Spectral Analysis

Synchronized, time-locked video and EEG and local field potential (LFP) signals were recorded using the Pinnacle system (Pinnacle Technology Inc., Lawrence, KS, United States). The LFP signals were amplified (100x) and digitized at a sampling frequency rate of 2000 Hz (high-pass filter 0.5 Hz and low-pass filter 500 Hz) and stored on a hard disk for offline analysis. The electrodes (one depth-coated tungsten in CMT (anteroposterior–AP: −1.35 mm; mediolateral–MD: 0; and dorsoventral–DV: −3.6 mm) and two screw-type cortical (AP: −1 mm; MD: ± 3 mm; DV: 0)) were implanted under continuous 1–1.5% isoflurane anesthesia. A screw electrode placed behind the lambda on each side of the midline served as the ground (right) and reference (left). Banamine® - Merck (i.p. 2.5 mg/kg) was applied right after surgery and every 24 h for 48 h. Seven to 10 days after surgery, mice of both strains (9 WT and 7 Ca_V_2.3 KO) were put in a recording chamber and EEG was recorded 15 min before the i.p. application of vehicle (25% 2-hydroxypropyl-β-cyclodextrin) or 80 mg/kg of 3β-OH as baseline recordings. EEG was recorded for 60 min under vehicle and under 80 mg/kg of 3β-OH. Although the LFP from CMT was recorded in all our *in vivo* experiments, only cortical EEG waveforms were analyzed for this study.

To compare spectra, 5 min of signal under 80 mg/kg of 3β-OH were extracted 60 min after 3β-OH injection. All spectral analyses were carried out using the and LabChart 8 and Origin 2018 software. The relative (%) power was calculated for different frequency ranges: δ (0.5–4 Hz), θ (4–8 Hz), α (8–13 Hz), β (13–30 Hz), and low γ (30–50 Hz).

After the completion of the experiments, mice were anesthetized with ketamine (100 mg/kg) and electrolytic lesions were made by passing 5 μA of current for 1 s (5 times). Mice were additionally anesthetized with isoflurane and perfused with ice-cold 0.1 M phosphate buffer containing 1% of potassium-ferrocyanide. The brains were extracted, kept in 4% paraformaldehyde (PFA) for 2 days, and sliced (100–150 μm) using a vibrating micro slicer (Leica VT 1200S). Images of coronal slices for electrode location conformation were obtained using a bright-field Zeiss stereoscope and the Zen Blue software.

### Drugs

Isoflurane was purchased from McKesson (San Francisco, CA) and 2-hydroxypropyl-β-cyclodextrin solution was purchased from Santa Cruz Biotechnology (Dallas, TX). Ketamine and Banamine® (Merck) were obtained from the pharmacy. All the other compounds were purchased from Sigma Chemical (St. Louis, MO). The 3β-OH was synthetized by the Doug Covey lab (Washington University School of Medicine, Saint Louis, MO, United States), as described elsewhere (Atluri et al., 2018). Neurosteroid 3β-OH was prepared as a 3 mM stock solution in dimethylsulfoxide (DMSO) for electrophysiological experiments; aliquots were stored at −20°C and diluted for use at a final concentration of 3 μM, which was delivered with a gravity-driven perfusion system. For EEG recordings and behavioral experiments, 3β-OH was dissolved in 25% of 2-hydroxypropyl-β-cyclodextrin solution and injected i.p.

### Data Analysis

In every *ex vivo* experiment, we attempted to obtain as many neurons as possible from each animal in order to minimize the number of animals used. Statistical analysis was performed using a two-way repeated measures (RM) ANOVA (in slice electrophysiology experiments, both factors were repeated) as well as Student’s unpaired and paired two-tailed t-tests where appropriate. Significance was accepted with *p* values <0.05. Statistical and graphical analyses were performed using the GraphPad Prism 9.0 software (GraphPad Software, La Jolla, CA, United States) and Origin 2018 (OriginLab, Northampton, MA, United States). All the EEG recordings were analyzed using LabChart 8 (ADInstruments, Dunedin, New Zealand).

## Results

### Reversible Inhibition of Recombinant Human Ca_V_2.3 Currents by 3β-OH

**FIGURE 1:**
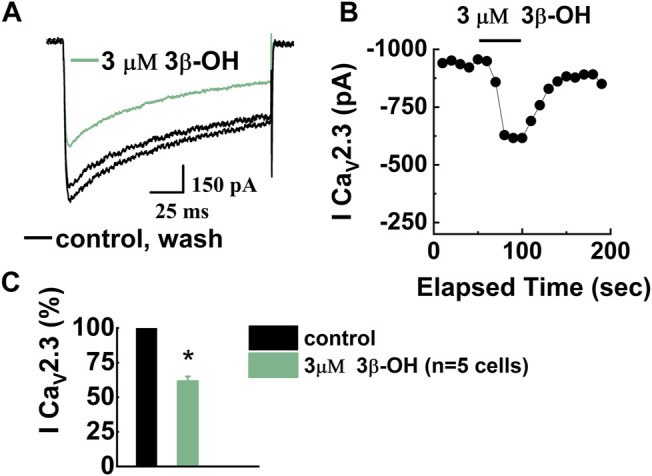
Inhibition of recombinant Ca_V_2.3 R-type current in HEK cells after bath application of 3β-OH. **(A)** Representative barium currents in control conditions before drug application (black), during the perfusion of 3 μM 3β-OH (green), and after drug washout (black). **(B)** Graph shows time course of reversible Ca_V_2.3 current amplitude reduction during the application of 3β-OH. **(C)** Bar graph shows the percentage of R-type current reduction during the perfusion of 3β-OH, which was averaged from multiple cells (*n* = 5, paired two-tailed *t*-test, *p* < 0.05).

We previously reported that 3 μM of 3β-OH inhibits T-currents in the rat TRN and CMT neurons in a voltage-dependent manner (Joksovic et al., 2007; Timic Stamenic et al., 2021), but its effect on R-type channels has not previously been studied. Hence, we first examined the effects of 3β-OH on isolated Ca_V_2.3 R-type currents in stably transfected HEK cells. Since we already investigated the effects of 3 μM 3β-OH on native neuronal Ca_V_3.1 T-channels and excitability (Timic Stamenic et al., 2021), here we wanted to explore effect of 3β-OH on recombinant R-type calcium channels using the same approach. In addition, we previously reported that 3 μM of 3β-OH is an IC_50_ for T-type calcium currents (Joksovic et al., 2007). Finally, our pharmacokinetic study in rats demonstrated that a concentration of 3 μM can be achieved in the brain during hypnosis with 3β-OH (Atluri et al., 2018). Therefore, we used 3 μM of 3β-OH for our *in vitro/ex vivo* electrophysiological recordings as a clinically relevant brain concentration in order to compare our new results with those of our previous studies. Representative barium current traces are presented in Figure 1A. Black traces are inward barium currents under control conditions and wash; green traces are from the same cell in the presence of 3 μM of 3β-OH. The time course of the current inhibition and the average data show that the addition of 3β-OH to the external solution reversibly reduced the R-type current by about 40% from the pre-drug baseline (Figures 1B,C, respectively).

### Selective Inhibition of R-type Currents by SNX-482 Reduced Tonic and Rebound Burst Firing in the Central Medial Nucleus of the Thalamus Neurons of Wild-Type Mice

**FIGURE 2:**
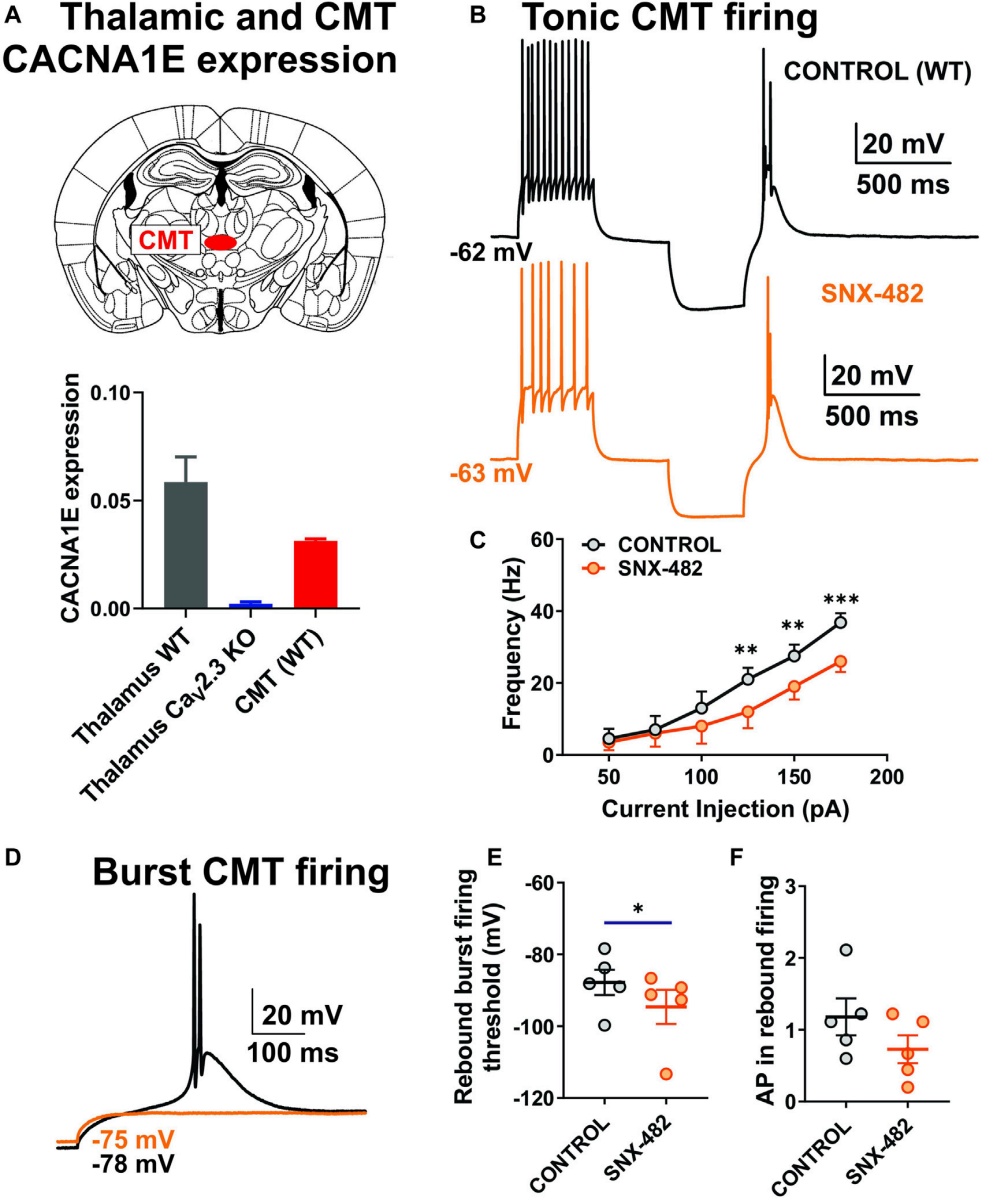
Stimulated tonic and rebound burst firing was reduced in thalamic neurons during the application of SNX-482 in WT animals. **(A)** Schematic presentation of CMT and qPCR data from the whole thalamus (WT and Ca_V_2.3 KO mice, *N* = 4 and 4, respectively) and CMT (WT animals, *N* = 3, technical replicate presented). **(B)** Representative traces of CMT action potential firing before (black) and during the application of 0.5 μM of SNX-482 (orange) recorded at an RMP of −60 mV. **(C)** Average frequency of stimulated tonic firing mode before and after the perfusion of SNX-482 (*n* = 5, two-way RM ANOVA: interaction F_5,20_ = 3.44, *p* = 0.021; SNX-482 F_1,4_ = 7.46, *p* = 0.052; current injection F_5,20_ = 40.73, *p* < 0.001, Šídák’s multiple comparisons test presented). **(D)** Representative traces of rebound burst firing before (black) and during the application of SNX-482 (orange) in the same CMT neuron; note that with the same current injection, there is no rebound burst firing after SNX-482 perfusion. **(E)** Rebound burst firing threshold after the hyperpolarization of the CMT neurons before and during the application of SNX-482 (*n* = 5, paired two-tailed *t*-test, t_4_ = 3.49, *p* = 0.025). **(F)** Average numbers of AP in rebound burst firing in control and after SNX-482 (*n* = 5, paired two-tailed *t*-test, t_4_ = 2.265, *p* = 0.086). *p < 0.05, **p < 0.01, ***p < 0.001.

Since previous studies only investigated the expression of the *cacna1e* gene in the whole thalamus, we used qPCR to assess Ca_V_2.3 expression in the CMT specifically. The top of Figure 2A shows a scheme of the coronal section of the rodent brain with the position of CMT (red dot), while the bottom of Figure 2A shows a summary graph of our qPCR experiments. Our data are consistent with the notion that mRNA for Ca_V_2.3 is significantly expressed in the whole thalamus and CMT in WT mice and completely absent from control experiments using tissue from KO animals. The role of R-type channels in synaptic transmission has been thoroughly studied; however, to the best of our knowledge, the role of these channels in different firing modes in CMT has never been demonstrated before. Hence, we next examined the effects of the use of 0.5 μM of SNX-482 on the output of CMT neurons using current-clamp recordings from acute brain slices in WT animals. This peptide was isolated from the venom of the African tarantula, *Hysterocrates gigas*, and identified as a potent and selective R-type channel inhibitor (Newcomb et al., 1998, 2000; Bourinet et al., 2001). Representative traces of APs before (black) and after SNX-482 (orange) application are shown in Figure 2B. We noticed that the applications of SNX-482 in multiple neurons significantly decreased the averaged stimulated tonic firing (Figure 2C) and increased the rebound burst firing threshold (Figure 2E), but did not significantly decrease the AP in rebound burst firing (Figure 2F). Representative traces of rebound burst firing in control conditions (black) and after SNX-482 (orange) are presented in Figure 2D. The RMP values did not differ before and after the drug application (mean ± SEM: −62.88 ± 1.27 mV in control group; −65.48 ± 2.26 mV after SNX-482 perfusion). Similarly, the values of input resistance (IR) at 100 pA current injection were not different between two groups (mean ± SEM: 306.9 ± 60.51 MΩ; 270.10 ± 50.81 MΩ, control and SNX-482 group, respectively).

### The 3β-OH Reduced Stimulated Tonic and Rebound Burst Firing in Central Medial Nucleus of the Thalamus Neurons in the Wild-Type Mice More Profoundly Than in Mutant Mice

**FIGURE 3:**
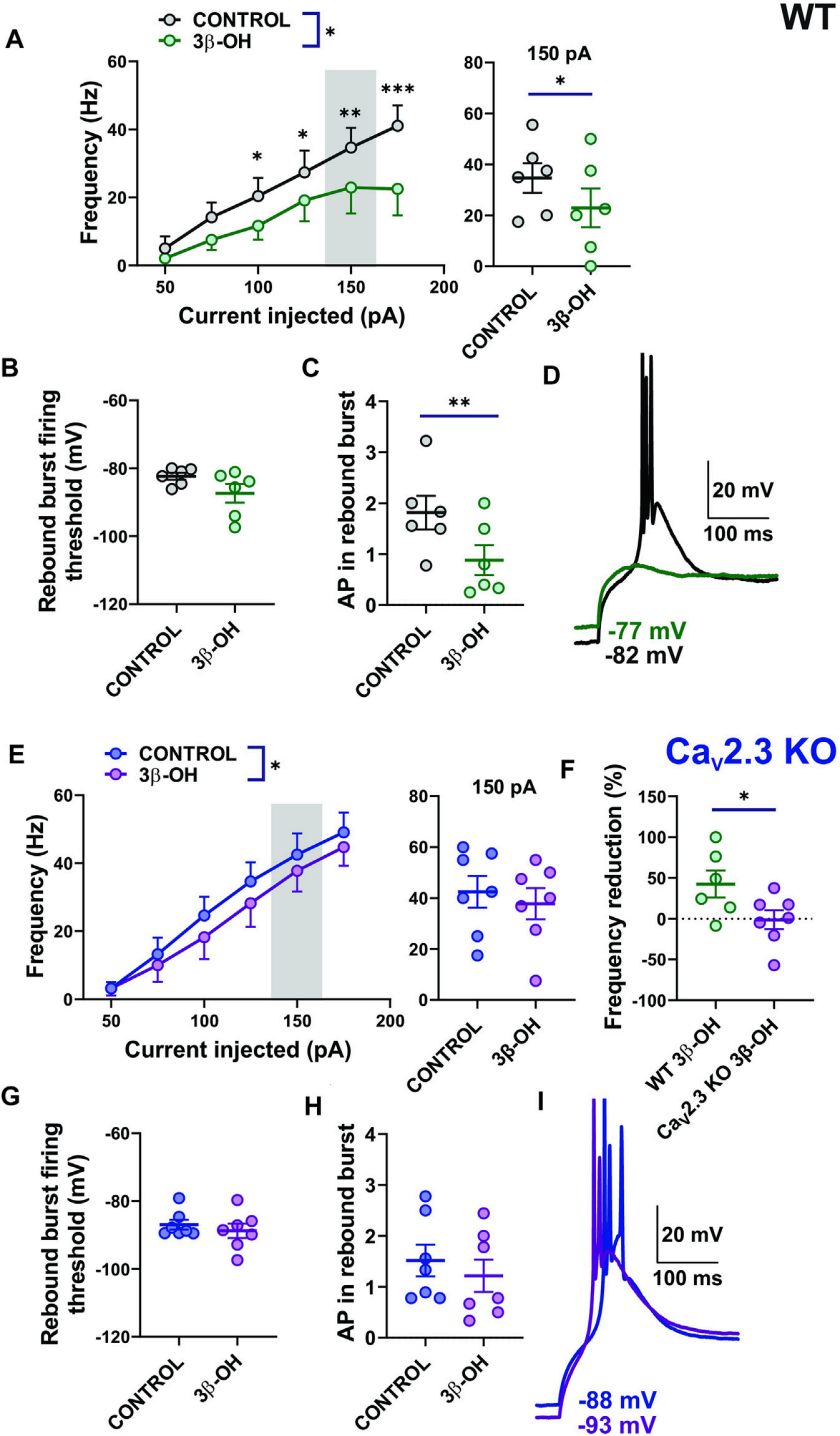
Stimulated tonic and rebound burst firing of CMT neurons were reduced in the WT group more profoundly than in the Ca_V_2.3 KO animals during the application of 3β-OH. **(A)** Average stimulated tonic frequency firing before (black) and during the application of 3 μM 3β-OH (green) across different current injection levels (50–175 pA) in thalamic slices from WT animals (*n* = 6, two-way RM ANOVA: interaction F_5,25_ = 3.40, *p* = 0.018; 3β-OH F_1,5_ = 10.27, *p* = 0.024; current injection F_5,25_ = 13.49, *p* < 0.001, Šídák’s multiple comparisons test presented), left; average tonic firing frequency at 150 pA current injection extracted from the same experiments (*n* = 6, paired two-tailed *t*-test t_5_ = 2.60, *p* = 0.048), right. **(B)** Rebound burst firing threshold after the hyperpolarization of the CMT neurons before and during the application of the neurosteroid in WT mice (*n* = 6, paired two-tailed *t*-test, t_5_ = 1.85, *p* = 0.124). **(C)** Average numbers of APs in rebound burst firing in control conditions and during the administration of 3β-OH in WT mice (*n* = 6, paired two-tailed *t*-test, t_5_ = 5.38, *p* = 0.003). **(D)** Representative traces of rebound burst firing before (black) and during the application of 3β-OH (green) in a WT mouse. Note that with the same current injection, there is no rebound burst firing after the perfusion of 3β-OH. **(E)** The average tonic firing frequency before (blue) and during the application of 3 μM of 3β-OH (purple) across different current injection (50–175 pA) in thalamic slices from Ca_V_2.3 KO animals (*n* = 7, two-way RM ANOVA: interaction F_5,30_ = 0.87, *p* = 0.510; 3β-OH F_1,6_ = 9.22, *p* = 0.023; current injection F_5,30_ = 42.80, *p* < 0.001), left panel. The average tonic frequency at 150 pA current injections from the same experiment (*n* = 7, paired two-tailed *t*-test t_6_ = 1.44, *p* = 0.199), right panel. **(F)** Average cumulative reduction in stimulated tonic firing normalized to baseline (%) in WT (*n* = 6) and Ca_V_2.3 KO (*n* = 7) animals, unpaired two-tailed *t*-test t_11_ = 2.21, *p* = 0.049. **(G)** Rebound burst firing threshold after the hyperpolarization of the CMT neurons in Ca_V_2.3 KO mice (*n* = 7, paired two-tailed *t*-test, t_6_ = 1.40, *p* = 0.210). **(H)** Average number of APs in rebound burst firing in control conditions and during the administration of 3β-OH in Ca_V_2.3 KO mice (*n* = 7, paired two-tailed *t*-test, t_6_ = 1.99, *p* = 0.093). **(I)** Representative traces of rebound burst firing before (blue trace) and during the application of 3β-OH (purple trace) in a Ca_V_2.3 KO mouse; note that with the same current injection, 3β-OH failed to abolish rebound burst firing. *p < 0.05, **p < 0.01, ***p < 0.001.

Since we demonstrated the inhibitory effect of selective R-type channel blocker SNX-482 on the CMT neurons’ excitability, we next examined the effect of 3 μM of 3β-OH on the stimulated tonic- and rebound burst firing mode of CMT neurons in WT and Ca_V_2.3 KO mice. We found that 3β-OH significantly reduced the frequency of APs over a wide range of current injections during the input–output protocols that we used to examine stimulated tonic firing mode in WT mice (Figure 3A, left). For example, 3β-OH strongly inhibited firing frequency by about 33% after a current injection of 150 pA (Figure 3A right). In contrast, although 3β-OH reduced the overall stimulated tonic firing frequency as measured by the input-output curves in the mutant mice to a smaller extent (Figure 3E left), it failed to significantly reduce the firing frequency elicited by a 150 pA current injection (Figure 3E right). Importantly, the normalized averaged frequency reduction across all current injections with 3β-OH was higher in WT (42.38%) in comparison to Ca_V_2.3 KO animals (mean value −1.44%, Figure 3F). In both WT and mutant animals, 3β-OH failed to lower the rebound burst firing threshold, though it did decrease the average AP number in the WT (Figures 3B,C) but not in the mutant animals (Figures 3G,H). Representative traces of rebound burst firing in the control conditions (black) and after 3β-OH application (green) recorded from WT animals are presented in Figure 3D. Representative traces of the rebound burst firing in control conditions (blue) and after 3β-OH application (purple) recorded from mutant mice are presented in Figure 3I. Furthermore, 3β-OH did not significantly affect the RMP or IR of the CMT neurons in WT mice or mice lacking a Ca_V_2.3 channel (data not shown).

In order to independently validate our findings with mutant animals, we next performed current-clamp recordings in CMT slices of the WT mice pretreated with 0.5 μM of SNX-482 (Supplementary Figure S1). The stimulated tonic firing slightly decreased after the perfusion of 3β-OH (Supplementary Figure S1A left), similarly to in Ca_V_2.3 KO animals (Figure 3A), with no significant effect on average tonic firing at 150 pA (Supplementary Figure S1A right). The average normalized frequency reduction achieved with 3β-OH recorded from untreated CMT neurons from WT mice, untreated Ca_V_2.3 KO animals, and WT mice with the addition of SNX-482 in the external solution are summarized in Figure S1B. The rebound burst firing threshold was not altered after 3β-OH perfusion (data not shown) and the average number of AP in rebound burst firing was not altered either (Supplementary Figure S1C). Additionally, we compared the baseline excitability of CMT neurons (stimulated tonic firing) in WT and Ca_V_2.3 KO animals and did not find any statistically significant difference between the two groups (data not shown), which could be due to compensatory changes in global KO animals. Importantly, we conclude that the pharmacological inhibition of the Ca_V_2.3 channel in WT mice largely diminishes the effects of 3β-OH on spike firing in CEM neurons, similar to the genetic deletion of this channel.

### The Ca_V_2.3 Channels Are Important for Neurosteroid-Induced Hypnosis

**FIGURE 4:**
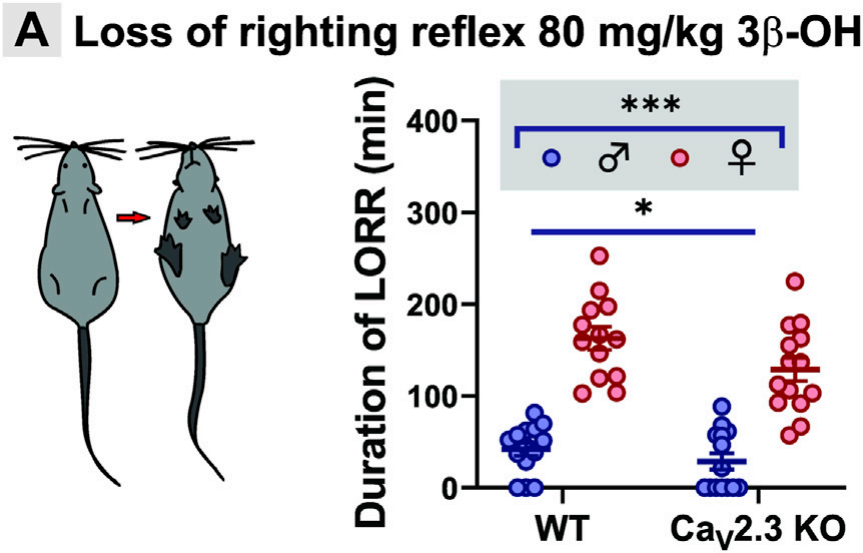
Sex- and genotype-dependent resistance to hypnosis measured by LORR in the Ca_V_2.3 KO mice when compared to the WT littermates after i.p. injections of 80 mg/kg 3β-OH. **(A)** Left, schematic presentation of LORR experiment; right, average duration of LORR after injections of 3β-OH (two-way ANOVA: interaction F_1,51_ = 0.99, *p* = 0.320; genotype F_1,51_ = 5.15, *p* = 0.027; sex F_1,51_ = 112.00, *p* < 0.001). *N* = 13–14 animals in WT group; *N* = 13–14 animals in Ca_V_2.3 KO group, *p < 0.05, ***p < 0.001.

Since we observed differences in thalamic excitability after the perfusion of 3β-OH in *ex vivo* slice recordings from WT and mutant animals, we next asked if Ca_V_2.3 channels are important for the hypnotic effect of this neurosteroid *in vivo*. We used an assay of LORR as a measure of the hypnotic potency of the drug, which was injected systemically into WT and mutant mice of both sexes. When mice were injected with 80 mg/kg of 3β-OH i.p., we observed sex differences in both WT and Ca_V_2.3 KO animals (Figure 4A), where females were more sensitive to the neurosteroid effect in comparison to males. For example, the average duration of LORR was about 3-fold longer in females in comparison to males in both WT and mutant cohorts. Importantly, the duration of LORR was about 25% longer in the WT group of animals in comparison to Ca_V_2.3 KO mice, indicating that mutant mice are relatively resistant to neurosteroid-induced hypnosis.

### Electroencephalogram Recordings Demonstrate Significant Oscillatory Differences Between WT and Ca_V_2.3 KO Mice During 3β-OH-Induced Unconsciousness in Female and Male Animals

**FIGURE 5:**
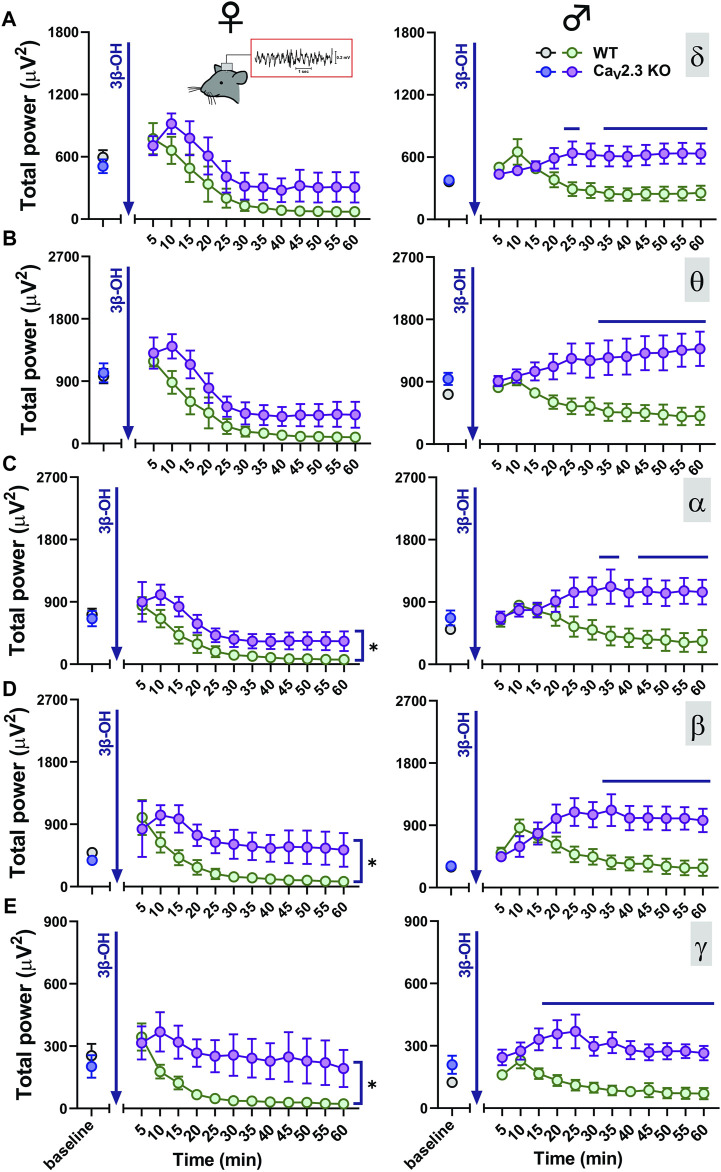
Total EEG power after i.p. injections of 80 mg/kg of 3β-OH during the time course of 60 min in WT (black, baseline; green, 3β-OH) and Ca_V_2.3 KO (blue, baseline; purple, 3β-OH) animals in the females is depicted on the left; in the males, this is presented on the right. Total δ **(A)**, θ **(B)**, α **(C)**, β **(D)**, and low γ **(E)** power after 3β-OH injection in females and males. **(A)** Left, two-way RM ANOVA: interaction F_11,88_ = 0.60, *p* = 0.823; genotype F_1,8_ = 3.39, *p* = 0.103; time F_11,88_ = 15.72, *p* < 0.001. **(B)** Left, two-way RM ANOVA: interaction F_11,88_ = 0.72, *p* = 0.715; genotype F_1,8_ = 4.57, *p* = 0.065; time F_11,88_ = 31.56, *p* < 0.001. **(C)** Left, two-way RM ANOVA: interaction F_11,88_ = 0.48, *p* = 0.910; genotype F_1,8_ = 6.64, *p* = 0.033; time F_11,88_ = 17.34, *p* < 0.001. **(D)** Left, two-way RM ANOVA: interaction F_11,88_ = 1.03, *p* = 0.430; genotype F_1,8_ = 9.54, *p* = 0.015; time F_11,88_ = 5.54, *p* < 0.001. **(E)** Left, two-way RM ANOVA: interaction F_11,88_ = 1.82, *p* = 0.065; genotype F_1,8_ = 7.94, *p* = 0.022; time F_11,88_ = 7.43, *p* < 0.001. **(A)** Right, two-way RM ANOVA: interaction F_11,99_ = 6.87, *p* < 0.001; genotype F_1,9_ = 7.05, *p* = 0.026; time F_11,99_ = 1.01, *p* = 0.443, Šídák’s multiple comparisons test presented on Figure as a line. **(B)** Right, two-way RM ANOVA: interaction F_11,99_ = 6.73, *p* < 0.001; genotype F_1,9_ = 7.72, *p* = 0.021; time F_11,99_ = 0.17, *p* = 0.999. Šídák’s multiple comparisons test presented as a solid black line. **(C)** Right, two-way RM ANOVA: interaction F_11,99_ = 6.85, *p* < 0.001; genotype F_1,9_ = 5.33, *p* = 0.046; time F_11,99_ = 0.87, *p* = 0.576. Šídák’s multiple comparisons test presented in figure as a line. **(D)** Right, two-way RM ANOVA: interaction F_11,99_ = 6.84, *p* < 0.001; genotype F_1,9_ = 6.25, *p* = 0.034; time F_11,99_ = 2.36, *p* = 0.012. Šídák’s multiple comparisons test presented in figure as a line. **(E)** Right, two-way RM ANOVA: interaction F_11,99_ = 3.64, *p* < 0.001; genotype F_1,9_ = 12.93, *p* = 0.006; time F_11,99_ = 4.31, *p* < 0.001. Šídák’s multiple comparisons test presented in figure as a line. *N* = 4-6 animals in WT group; *N* = 5–7 animals in Ca_V_2.3 KO group.

We next tested the hypothesis that Ca_V_2.3 channels may have an effect on the 3β-OH-induced thalamocortical rhythmic oscillations. We used cortical EEG recordings in WT and Ca_V_2.3 KO mice to investigate if Ca_V_2.3 channels are important for oscillations *in vivo* during systemic administration of 80 mg/kg 3β-OH in female and male mice. After the injection of 80 mg/kg of 3β-OH i.p., we noticed a transient rise (5–10 min after neurosteroid injection) in total power in all analyzed frequencies followed by a global EEG reduction in all groups except male mutant mice (Figure 5). During analyzed period of 60 min, we observed statistically significant differences between WT and mutant male animals in all oscillations (Figures 5A–E right, horizontal line). Although differences were statistically significant only for oscillations in the α, β and low γ bands (Figures 5C–E left), WT female animals showed more EEG reduction in comparison to mutant females during 60 min of recordings following injections of 3β-OH. We did not observed baseline differences in any of the tested frequencies between the groups.

**FIGURE 6:**
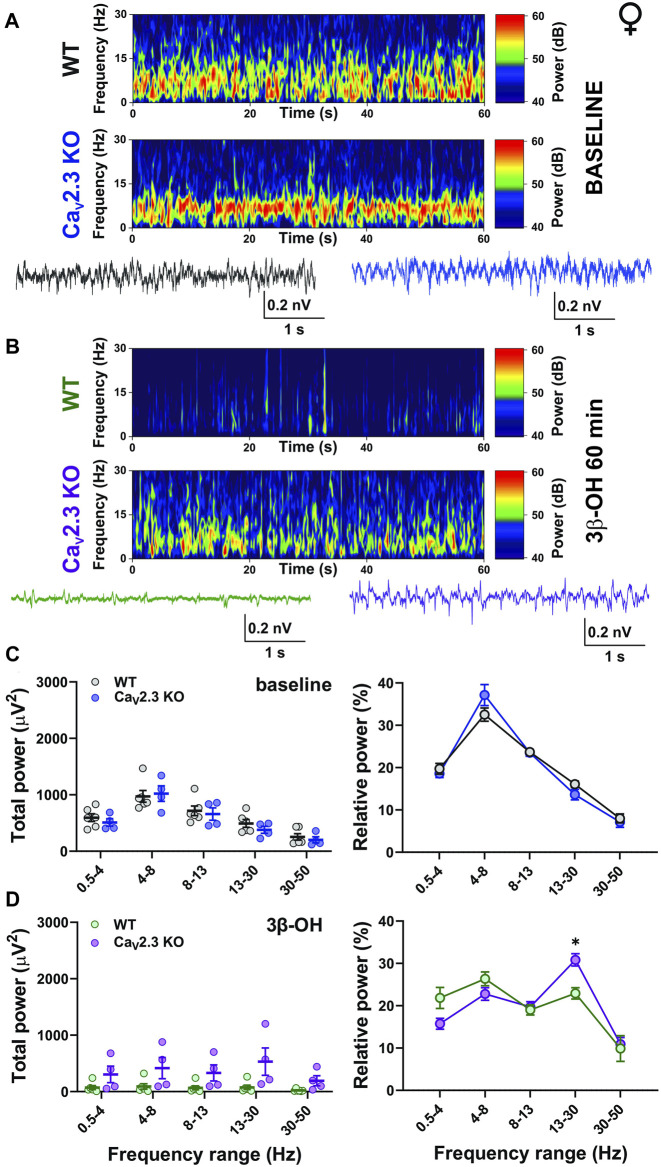
The effects of 3β-OH on EEG in female WT and Ca_V_2.3 KO animals at a time point of 60 min after i.p. injections of 80 mg/kg of 3β-OH. **(A)** Representative baseline heat map from a female WT (top) and a Ca_V_2.3 KO (bottom) animal with the representative EEG traces (WT, black; Ca_V_2.3 KO, blue). **(B)** Representative heat map from a female WT (top) and a Ca_V_2.3 KO (bottom) animal with the representative EEG traces (WT, green; Ca_V_2.3 KO, purple) at time point of 60 min after injections of 3β-OH. **(C)** Total power during baseline (left) and relative baseline power (right). **(D)** Total power (left) and relative power (right, two-way RM ANOVA: interaction F_4,32_ = 2.90, *p* = 0.004; genotype F_1,8_ = 0.09, *p* = 0.771; frequency F_4,32_ = 16.44, *p* < 0.001. Šídák’s multiple comparisons test presented) at 60 min after 3β-OH. *N* = 6 animals in WT group; *N* = 4 animals in Ca_V_2.3 KO group, *p < 0.05.

**FIGURE 7:**
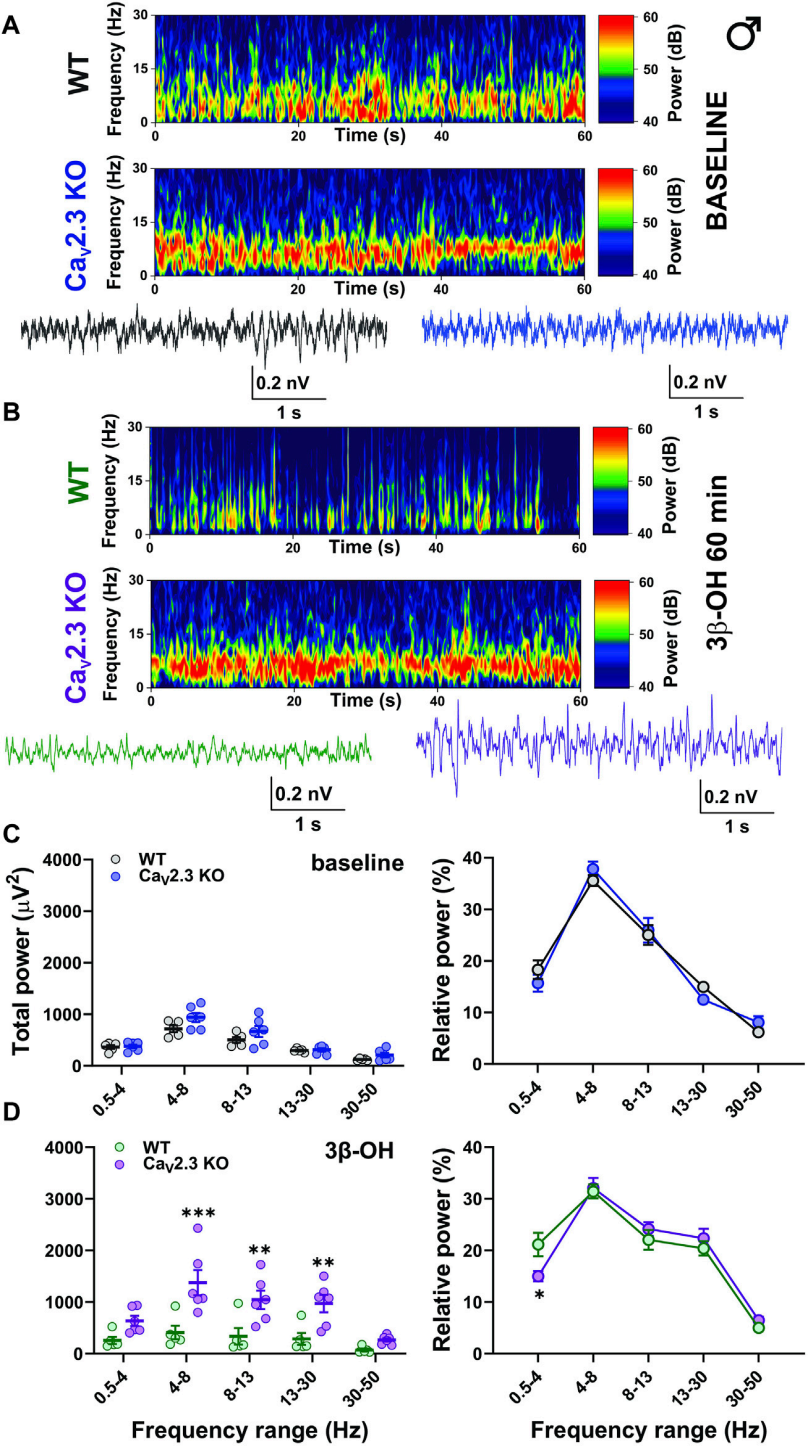
The effects of 3β-OH on EEG in male WT and Ca_V_2.3 KO animals at the time point of 60 min after i.p. injections of 80 mg/kg of 3β-OH. **(A)** Representative baseline heat map from a male WT (top) and a Ca_V_2.3 KO (bottom) animal with the representative EEG traces (WT, black; Ca_V_2.3 KO, blue). **(B)** Representative heat map from a male WT (top) and a Ca_V_2.3 KO (bottom) animal with the representative EEG traces (WT, green; Ca_V_2.3 KO, purple) 60 min after 3β-OH. **(C)** Total power during baseline (left) and relative baseline power (right). **(D)** Total power (left, two-way RM ANOVA: interaction F_4,36_ = 5.86, *p* = 0.001; genotype F_1,9_ = 11.49, *p* = 0.008; frequency F_4,36_ = 19.49, *p* < 0.001, Šídák’s multiple comparisons test presented) and relative power (left, two-way RM ANOVA: interaction F_4,36_ = 2.09, *p* = 0.103; genotype F_1,9_ = 1.84, *p* = 0.208; frequency F_4,36_ = 61.56, *p* < 0.001, Šídák’s multiple comparisons test presented) 60 min after 3β-OH. *N* = 5 animals in WT group and *N* = 6 animals in Ca_V_2.3 KO group. *p < 0.05, **p < 0.01, ***p < 0.001.

Next, we performed a detailed analysis of oscillatory differences *in vivo* between WT and Ca_V_2.3 KO female and male animals 60 min after i.p. injections of 80 mg/kg of 3β-OH (Figures 6, 7, respectively). Representative cortical spectrograms under baseline conditions and after the administration of 3β-OH recorded from WT and Ca_V_2.3 KO female mice with representative EEG traces are presented in Figures 6A,B. The analysis of the total and relative baseline power did not show any differences between WT and mutant animals (Figure 6C, left and right). Similarly, the analysis of total power 60 min after neurosteroid injection did not reveal differences between the two groups of animals (Figure 6D, left). In contrast, the analysis of relative power after the administration of 3β-OH revealed differences in the β frequency range, with an increase in β oscillations in mutant female mice of about 30% compared to the WT group (Figure 6C, right).

Representative cortical spectrograms recorded from WT and Ca_V_2.3 KO male mice with representative EEG traces under baseline conditions and 3β-OH are presented in Figures 7A,B. The analysis of the total and relative baseline power did not show differences between the WT and mutant animals (Figure 7C, left and right). However, the analysis of total power recorded 60 min after the injection of 3β-OH revealed a higher EEG power in θ, α, and β oscillations in mutant male mice in comparison to WT animals (Figure 7C, left). Additionally, the analysis of relative power revealed a significant rise in slow oscillations in the δ frequency range in WT compared to Ca_V_2.3 KO male mice (Figure 7D, right).

## Disscusion

Here, we first investigated the effects of 3β-OH on isolated recombinant Ca_V_2.3 R-type current expressed in HEK-293 cells. We found that the R-type current was reversibly reduced by about 40% after the perfusion of 3 μM of 3β-OH, indicating the ability of this neurosteroid to inhibit the Ca_V_2.3 channel in a relevant brain concentration during hypnosis, as determined by LORR (Atluri et al., 2018). In order to further investigate the role of R-type channels in CMT neuronal excitability, we performed *ex vivo* electrophysiological recordings and found a reduction in stimulated tonic firing after the application of SNX-482, a selective R-type channel inhibitor (Newcomb et al., 1998; 2000; Bourinet et al., 2001). Additionally, we found that the threshold for rebound burst generation was increased after the application of SNX-482, indicating the need for greater neuronal hyperpolarization in order to generate rebound bursting when R-type channels are blocked. Similarly, we studied the effect of 3β-OH on neuronal excitability in CMT in both WT and mutant animals. Predictably, we found a profound reduction in tonic and rebound burst firing in CMT neurons from WT animals after the administration of 3β-OH and a modest decrease in stimulated tonic firing in CMT neurons from Ca_V_2.3 KO mice and CMT neurons from WT mice that had been pretreated with SNX-482. Since we previously reported the ability of 3β-OH to block Ca_V_3.1 channels in CMT neurons (Timic Stamenic et al., 2021), the reduction in excitability in mutant animals is also related to their inhibitory effect on T-type calcium channels. Furthermore, the existence of compensatory changes and the possible upregulation of other VGCC (mostly T-type calcium channels) in Ca_V_2.3 mutant mice cannot be excluded. However, previous studies did not find any change in the thalamic T-channel expression levels in Ca_V_2.3 KO animals in comparison to their WT littermates (Weiergräber et al., 2006; Siwek et al., 2014).

Next, we examined the involvement of R-type channels in the hypnotic effects of neurosteroids using i.p. injections of 80 mg/kg of 3β-OH to produce LORR in WT and mutant animals of both sexes. Sex differences from the Ca_V_2.3 channel had been observed earlier in the mice model of central sensitization, where Ca_V_2.3 inhibition, especially in female mice, had a significant effect (Ferreira et al., 2021). Furthermore, sex differences were demonstrated after the application of neuroactive steroids in a variety of rodent models (Fink et al., 1982; Arenillas and Gomez de Segura, 2018; Joksimovic et al., 2021). We recently reported the sex-dependent hypnotic effect of 3β-OH in rats (Joksimovic et al., 2021); thus, here, we confirmed the sex-dependent effect of 3β-OH in Ca_V_2.3 KO and WT animals. Consistent with our rat study, we found that female mice had longer LORR durations in comparison to male animals. Importantly, WT animals were more sensitive to 3β-OH-induced hypnosis than Ca_V_2.3 KO mice, strongly suggesting that the inhibition of Ca_V_2.3 channels at least partly contributes to the hypnosis induced by 3β-OH in mice in both sexes.

It is known that the Cav2.3 channel activation requires the use of a mid to high voltage range, which is higher than for T-type calcium channels but lower than for other HVA channels (Catterall et al., 2005). Hence, the activation of these channels in thalamic neurons can facilitate the tonic firing mode and blockade can decrease the excitability and alter the generation of thalamocortical oscillations. It is known that many general anesthetics may generate rhythmic oscillations of bursts with blackout sequences (suppressions), and this burst-suppression pattern is generally thought to correlate with the blockage of thalamocortical sensory information (Steriade et al., 1994). We previously demonstrated that presynaptic R-type channels contribute to isoflurane-induced effects on inhibitory synaptic transmission in NRT and cortical EEG burst-suppression patterns (Joksovic et al., 2009). Additionally, it has been demonstrated that Ca_V_2.3-deficient mice have a reduced wake duration and increased duration of slow-wave natural and urethane-induced sleep (SWS) (Siwek et al., 2014). Thus, we performed detailed EEG analyses after neurosteroid injection in adult Ca_V_2.3 KO mice and their WT littermates. Although i.p. injections of neurosteroids rarely show the characteristic burst-suppression patterns of volatile anesthetics, we observed a drastic reduction in EEG power predominantly in female WT animals and a lack of EEG power reduction in mutant male mice (Figures 5–7). It is not surprising that voltage-gated calcium channels have relevance in sleep physiology and epileptogenesis due to their unique electrophysiological properties and cellular distribution (Talley et al., 1999; Lee et al., 2004; Anderson et al., 2005; Weiergräber et al., 2008; Siwek et al., 2014). Hence, we reasoned that Ca_V_2.3 channels might be involved in drug-induced hypnotic/anesthetic effects. Recent studies have demonstrated the important role of Ca_V_2.3 calcium channels in thalamocortical rhythmicity (Weiergräber et al., 2006, 2008; Zaman et al., 2011) and in septohippocampal synchronization associated with θ oscillations (Müller et al., 2012). Over time, we showed that there is a difference in total α, β, and low γ oscillations between WT and Ca_V_2.3 KO female animals, with a more profound reduction in these frequencies seen in WT mice after neurosteroid administration. A similar effect was seen in the male mice, where 3β-OH showed the reduction of total power in all analyzed frequencies in WT animals but failed to do so in mutant males. Due to this, the differences in neurosteroid effect seen in the EEG oscillations of WT and KO male animals over time appeared more dramatic than those in females.

Consistent with the observed effects on LORR *in vivo*, our EEG recordings revealed different effects of 3β-OH in WT and Ca_V_2.3 KO mice, with the presence of a sex-dependent effect. For example, the relative δ power 60 min after the application of 3β-OH was higher in WT male mice compared to mutant mice, reflecting the different behavioral states of the animals. Additionally, the analysis of the total power under the neurosteroid revealed the inability of the 3β-OH to suppress EEG power in male mutant animals. On the contrary, the total power at a time point of 60 min after the application of 3β-OH did not differ between the mutant and control female animals, but the relative power showed a stronger increase in β oscillations in mutant animals. The relative δ oscillations were somewhat higher in WT than in mutant female mice, although this effect was not significant. It is well known that during light hypnotic/sedative states, an increase in β oscillations is frequently seen, and during deep hypnosis an increase in slow δ frequencies, burst-suppression activity, and EEG reduction are present (Stamenic and Todorovic, 2022). Hence, we posit that the lower relative δ values in mutants and higher relative β power in mutant females implied the inability of the neurosteroid to induce a deeper hypnotic state in Ca_V_2.3 KO mice. Interestingly, we did not observed baseline EEG differences between the WT and Ca_V_2.3 KO animals.

The inability of 3β-OH to block thalamic rebound bursting, suppress EEG, and increase slow-frequency oscillation in Ca_V_2.3 KO male mice is likely to explain the inability of the neurosteroid to effectively induce hypnosis in these animals. The LORR and EEG changes observed in female animals suggest the existence of the same neurosteroid-induced mechanisms in WT and mutant mice, with stronger 3β-OH-induced hypnosis and EEG reduction seen in female WT animals. The observed inhibitory effects on stimulated tonic firing in both mutant and control cohorts, even though more prominent in WT mice, could also implicate the involvement of the Ca_V_3.1 channel, which works in concert with the inhibition of the Ca_V_2.3 channel in the thalamus as a target for 3β-OH’s effects. However, it appears that the reduced tonic excitability seen in many mutant male animals could not effectively induce hypnosis. Although the interpretation of behavioral studies using global KO mice may be complicated due to possible compensatory changes, our results achieved with Ca_V_2.3 KO animals strongly suggest that our approach is productive for validating, for the first time, the role of the R-type channel in neurosteroid-induced hypnosis.

## Data Availability

The original contributions presented in the study are included in the article/Supplementary Material, further inquiries can be directed to the corresponding author.

## References

[B2] Anderson M. P. Mochizuki T. Xie J. Fischler W. Manger J. P. Talley E. M. (2005). Thalamic CaV3.1 T-type Ca^2+^ Channel Plays a Crucial Role in Stabilizing Sleep. Proc. Natl. Acad. Sci. U. S. A. 102, 1743–1748. 10.1073/pnas.0409644102 15677322 PMC547889

[B3] Arenillas M. Gomez de Segura I. A. (2018). Anaesthetic Effects of Alfaxalone Administered Intraperitoneally Alone or Combined with Dexmedetomidine and Fentanyl in the Rat. Lab. Anim. 52, 588–598. 10.1177/0023677218764214 29580166

[B4] Atluri N. Joksimovic S. M. Oklopcic A. Milanovic D. Klawitter J. Eggan P. (2018). A Neurosteroid Analogue with T-type Calcium Channel Blocking Properties Is an Effective Hypnotic, but Is Not Harmful to Neonatal Rat Brain. Br. J. Anaesth. 120, 768–778. 10.1016/J.BJA.2017.12.039 29576117 PMC6200096

[B5] Baker R. Gent T. C. Yang Q. Parker S. Vyssotski A. L. Wisden W. (2014). Altered Activity in the Central Medial Thalamus Precedes Changes in the Neocortex during Transitions into Both Sleep and Propofol Anesthesia. J. Neurosci. 34, 13326–13335. 10.1523/JNEUROSCI.1519-14.2014 25274812 PMC4180471

[B6] Belelli D. Lambert J. J. (2005). Neurosteroids: Endogenous Regulators of the GABA(A) Receptor. Nat. Rev. Neurosci. 6, 565–575. 10.1038/nrn1703 15959466

[B7] Benkert J. Hess S. Roy S. Beccano-Kelly D. Wiederspohn N. Duda J. (2019). Cav2.3 Channels Contribute to Dopaminergic Neuron Loss in a Model of Parkinson's Disease. Nat. Commun. 10, 5094–5111. 10.1038/s41467-019-12834-x 31704946 PMC6841684

[B8] Berecki G. Motin L. Adams D. J. (2016). Voltage-Gated R-type Calcium Channel Inhibition via Human μ-, δ-, and κ-opioid Receptors Is Voltage-Independently Mediated by Gβγ Protein Subunits. Mol. Pharmacol. 89, 187–196. 10.1124/MOL.115.101154 26490245

[B9] Bourinet E. Stotz S. C. Spaetgens R. L. Dayanithi G. Lemos J. Nargeot J. (2001). Interaction of SNX482 with Domains III and IV Inhibits Activation Gating of alpha(1E) (Ca(V)2.3) Calcium Channels. Biophys. J. 81, 79–88. 10.1016/S0006-3495(01)75681-0 11423396 PMC1301493

[B10] Breustedt J. Vogt K. E. Miller R. J. Nicoll R. A. Schmitz D. (2003). Alpha1E-containing Ca^2+^ Channels Are Involved in Synaptic Plasticity. Proc. Natl. Acad. Sci. U. S. A. 100, 12450–12455. 10.1073/pnas.2035117100 14519849 PMC218778

[B11] Catterall W. A. Perez-Reyes E. Snutch T. P. Striessnig J. (2005). International Union of Pharmacology. XLVIII. Nomenclature and Structure-Function Relationships of Voltage-Gated Calcium Channels. Pharmacol. Rev. 57, 411–425. 10.1124/PR.57.4.5 16382099

[B12] Catterall W. A. (2011). Voltage-Gated Calcium Channels. Cold Spring Harb. Perspect. Biol. 3, a003947–23. 10.1101/CSHPERSPECT.A003947 21746798 PMC3140680

[B13] Dietrich D. Kirschstein T. Kukley M. Pereverzev A. Von Der Brelie C. Schneider T. (2003). Functional Specialization of Presynaptic Cav2.3 Ca^2+^ Channels. Neuron 39, 483–496. 10.1016/S0896-6273(03)00430-6 12895422

[B15] Ferreira M. A. Lückemeyer D. D. Macedo-Júnior S. J. Schran R. G. Silva A. M. Prudente A. S. (2021). Sex-dependent Cav2.3 Channel Contribution to the Secondary Hyperalgesia in a Mice Model of Central Sensitization. Brain Res. 1764, 147438. 10.1016/J.BRAINRES.2021.147438 33753067

[B16] Fink G. Sarkar D. K. Dow R. C. Dick H. Borthwick N. Malnick S. (1982). Sex Difference in Response to Alphaxalone Anaesthesia May Be Oestrogen Dependent. Nature 298, 270–272. 10.1038/298270a0 7201079

[B19] Helbig K. L. Lauerer R. J. Bahr J. C. Souza I. A. Myers C. T. Uysal B. (2018). De Novo Pathogenic Variants in CACNA1E Cause Developmental and Epileptic Encephalopathy with Contractures, Macrocephaly, and Dyskinesias. Am. J. Hum. Genet. 103, 666–678. 10.1016/J.AJHG.2018.09.006 30343943 PMC6216110

[B20] Jeong J. Y. Kweon H. J. Suh B. C. (2016). Dual Regulation of R-type CaV2.3 Channels by M1 Muscarinic Receptors. Mol. Cells 39, 322–329. 10.14348/MOLCELLS.2016.2292 26923189 PMC4844939

[B21] Joksimovic S. M. Sampath D. Krishnan K. Covey D. F. Jevtovic-Todorovic V. Raol Y. H. (2021). Differential Effects of the Novel Neurosteroid Hypnotic (3β,5β,17β)-3-Hydroxyandrostane-17-Carbonitrile on Electroencephalogram Activity in Male and Female Rats. Br. J. Anaesth. 127, 435–446. 10.1016/J.BJA.2021.03.029/ATTACHMENT/B2F1CD94-8A98-4D87-8934-09AB758B4B9B/MMC1 33972091 PMC8451239

[B22] Joksovic P. M. Covey D. F. Todorovic S. M. (2007). Inhibition of T-type Calcium Current in the Reticular Thalamic Nucleus by a Novel Neuroactive Steroid. Ann. N. Y. Acad. Sci. 1122, 83–94. 10.1196/annals.1403.006 18077566

[B23] Joksovic P. M. Weiergräber M. Lee W. Struck H. Schneider T. Todorovic S. M. (2009). Isoflurane-Sensitive Presynaptic R-type Calcium Channels Contribute to Inhibitory Synaptic Transmission in the Rat Thalamus. J. Neurosci. 29, 1434–1445. 10.1523/JNEUROSCI.5574-08.2009 19193890 PMC2659547

[B24] Kamp M. A. Krieger A. Henry M. Hescheler J. Weiergräber M. Schneider T. (2005). Presynaptic 'Ca2.3-containing' E-type Ca Channels Share Dual Roles during Neurotransmitter Release. Eur. J. Neurosci. 21, 1617–1625. 10.1111/J.1460-9568.2005.03984.X 15845089

[B25] Kolmac C. I. Mitrofanis J. (1997). Organisation of the Reticular Thalamic Projection to the Intralaminar and Midline Nuclei in Rats. J. Comp. Neurol. 377, 165–178. 10.1002/(SICI)1096-9861(19970113)377:2<165::AID-CNE2>3.0 8986879

[B26] Lambert J. J. Belelli D. Hill-Venning C. Peters J. A. (1995). Neurosteroids and GABAA Receptor Function. Trends Pharmacol. Sci. 16, 295–303. 10.1016/S0165-6147(00)89058-6 7482994

[B27] Lau C. Ranasinghe M. G. Shiels I. Keates H. Pasloske K. Bellingham M. C. (2013). Plasma Pharmacokinetics of Alfaxalone after a Single Intraperitoneal or Intravenous Injection of Alfaxan(®) in Rats. J. Vet. Pharmacol. Ther. 36, 516–520. 10.1111/JVP.12055 23600373

[B28] Lee J. Kim D. Shin H. S. (2004). Lack of Delta Waves and Sleep Disturbances during Non-rapid Eye Movement Sleep in Mice Lacking alpha1G-Subunit of T-type Calcium Channels. Proc. Natl. Acad. Sci. U. S. A. 101, 18195–18199. 10.1073/pnas.0408089101 15601764 PMC539778

[B30] Metz A. E. Jarsky T. Martina M. Spruston N. (2005). R-type Calcium Channels Contribute to Afterdepolarization and Bursting in Hippocampal CA1 Pyramidal Neurons. J. Neurosci. 25, 5763–5773. 10.1523/JNEUROSCI.0624-05.2005 15958743 PMC6724888

[B31] Müller R. Struck H. Ho M. S. Brockhaus-Dumke A. Klosterkötter J. Broich K. (2012). Atropine-sensitive Hippocampal θ Oscillations Are Mediated by Cav2.3 R-type Ca²⁺ Channels. Neuroscience 205, 125–139. 10.1016/J.NEUROSCIENCE.2011.12.032 22240250

[B32] Nakashima Y. M. Todorovic S. M. Pereverzev A. Hescheler J. Schneider T. Lingle C. J. (1998). Properties of Ba^2+^ Currents Arising from Human α1E and α1Eβ3 Constructs Expressed in HEK293 Cells: Physiology, Pharmacology, and Comparison to Native T-type Ba2+ Currents. Neuropharmacology 37, 957–972. 10.1016/S0028-3908(98)00097-5 9833625

[B33] Neumaier F. Alpdogan S. Hescheler J. Schneider T. (2018). Protein Phosphorylation Maintains the Normal Function of Cloned Human Cav2.3 Channels. J. Gen. Physiol. 150, 491–510. 10.1085/JGP.201711880 29453293 PMC5839719

[B34] Neumaier F. Schneider T. Albanna W. (2020). Cav2.3 Channel Function and Zn^2+^-Induced Modulation: Potential Mechanisms and (Patho)physiological Relevance. Channels (Austin) 14, 362–379. 10.1080/19336950.2020.1829842 33079629 PMC7583514

[B35] Newcomb R. Szoke B. Palma A. Wang G. Chen Xh. Hopkins W. (1998). Selective Peptide Antagonist of the Class E Calcium Channel from the Venom of the Tarantula Hysterocrates Gigas. Biochemistry 37, 15353–15362. 10.1021/bi981255g 9799496

[B36] Newcomb R. Chen X.-h. Dean R. Dayanithi G. Cong R. Szoke B. (2000). SNX-482: A Novel Class E Calcium Channel Antagonist from Tarantula Venom. CNS Drug Rev. 6, 153–173. 10.1111/j.1527-3458.2000.tb00143.x

[B37] Ortner N. J. (2018). Voltage-Gated Ca2^+^ Channels in Dopaminergic Substantia Nigra Neurons: Therapeutic Targets for Neuroprotection in Parkinson's Disease? Front. Synaptic Neurosci. 13, 5. 10.3389/FNSYN.2021.636103 PMC7952618 33716705

[B38] Parajuli L. K. Nakajima C. Kulik A. Matsui K. Schneider T. Shigemoto R. (2012). Quantitative Regional and Ultrastructural Localization of the Ca(v)2.3 Subunit of R-type Calcium Channel in Mouse Brain. J. Neurosci. 32, 13555–13567. 10.1523/JNEUROSCI.1142-12.2012 23015445 PMC6621359

[B39] Pereverzev A. Mikhna M. Vajna R. Gissel C. Henry M. Weiergräber M. (2002). Disturbances in Glucose-Tolerance, Insulin-Release, and Stress-Induced Hyperglycemia upon Disruption of the Ca(v)2.3 (Alpha 1E) Subunit of Voltage-Gated Ca(2+) Channels. Mol. Endocrinol. 16, 884–895. 10.1210/MEND.16.4.0801 11923483

[B40] Ricoy U. M. Frerking M. E. (2014). Distinct Roles for Cav2.1-2.3 in Activity-dependent Synaptic DynamicsJ. Neurophysiol. 111, 2404–2413. 10.1152/JN.00335.2013/ASSET/IMAGES/LARGE/Z9K0091423960007 PMC4044429 24523520

[B41] Rupprecht R. (2003). Neuroactive Steroids: Mechanisms of Action and Neuropsychopharmacological Properties. Psychoneuroendocrinology 28, 139–168. 10.1016/S0306-4530(02)00064-1 12510009

[B42] Saegusa H. Kurihara T. Zong S. Minowa O. Kazuno A. Han W. (2000). Altered Pain Responses in Mice Lacking Alpha 1E Subunit of the Voltage-dependent Ca2+ Channel. Proc. Natl. Acad. Sci. U. S. A. 97, 6132–6137. 10.1073/PNAS.100124197 10801976 PMC18570

[B43] Saegusa H. Matsuda Y. Tanabe T. (2002). Effects of Ablation of N- and R-type Ca(^2+^) Channels on Pain Transmission. Neurosci. Res. 43, 1–7. 10.1016/S0168-0102(02)00017-2 12074836

[B44] Scheibel M. E. Scheibel A. B. (1966). The Organization of the Nucleus Reticularis Thalami: A Golgi Study. Brain Res. 1, 43–62. 10.1016/0006-8993(66)90104-1 4160184

[B45] Schneider T. Alpdogan S. Hescheler J. Neumaier F. (2018). *In Vitro* and *In Vivo* Phosphorylation of the Cav2.3 Voltage-Gated R-type Calcium Channel. Channels (Austin) 12, 326–334. 10.1080/19336950.2018.1516984 30165790 PMC6986797

[B46] Schneider T. Neumaier F. Hescheler J. Alpdogan S. (2020). Cav2.3 R-type Calcium Channels: from its Discovery to Pathogenic De Novo CACNA1E Variants: a Historical Perspective. Pflugers Arch. 472, 811–816. 10.1007/S00424-020-02395-0/FIGURES/2 32529299 PMC7351833

[B48] Shcheglovitov A. Vitko I. Lazarenko R. M. Orestes P. Todorovic S. M. Perez-Reyes E. (2012). Molecular and Biophysical Basis of Glutamate and Trace Metal Modulation of Voltage-Gated Ca(v)2.3 Calcium Channels. J. Gen. Physiol. 139, 219–234. 10.1085/JGP.201110699 22371363 PMC3289959

[B49] Simms B. A. Zamponi G. W. (2014). Neuronal Voltage-Gated Calcium Channels: Structure, Function, and Dysfunction. Neuron 82, 24–45. 10.1016/j.neuron.2014.03.016 24698266

[B50] Siwek M. E. Müller R. Henseler C. Broich K. Papazoglou A. Weiergräber M. (2014). The CaV2.3 R-type Voltage-Gated Ca2^+^ Channel in Mouse Sleep Architecture. Sleep 37, 881–892. 10.5665/SLEEP.3652 24790266 PMC3985108

[B51] Soong T. W. Stea A. Hodson C. D. Dubel S. J. Vincent S. R. Snutch T. P. (1993). Structure and Functional Expression of a Member of the Low Voltage-Activated Calcium Channel Family. Science 260, 1133–1136. 10.1126/SCIENCE.8388125 8388125

[B52] Stell B. M. Brickley S. G. Tang C. Y. Farrant M. Mody I. (2003). Neuroactive Steroids Reduce Neuronal Excitability by Selectively Enhancing Tonic Inhibition Mediated by Delta Subunit-Containing GABAA Receptors. Proc. Natl. Acad. Sci. U. S. A. 100, 14439–14444. 10.1073/pnas.2435457100 14623958 PMC283610

[B53] Steriade M. Amzica F. Contreras D. (1994). Cortical and Thalamic Cellular Correlates of Electroencephalographic Burst-Suppression. Electroencephalogr. Clin. Neurophysiol. 90, 1–16. 10.1016/0013-4694(94)90108-2 7509269

[B54] Steriade M. Parent A. Hada J. (1984). Thalamic Projections of Nucleus Reticularis Thalami of Cat: A Study Using Retrograde Transport of Horseradish Peroxidase and Fluorescent Tracers. J. Comp. Neurol. 229, 531–547. 10.1002/cne.902290407 6209310

[B55] Talley E. M. Cribbs L. L. Lee J. H. Daud A. Perez-Reyes E. Bayliss D. A. (1999). Differential Distribution of Three Members of a Gene Family Encoding Low Voltage-Activated (T-type) Calcium Channels. J. Neurosci. 19, 1895–1911. 10.1523/jneurosci.19-06-01895.1999 10066243 PMC6782581

[B56] Timic Stamenic T. Feseha S. Manzella F. M. Wallace D. Wilkey D. Corrigan T. (2021). The T-type Calcium Channel Isoform Cav3.1 Is a Target for the Hypnotic Effect of the Anaesthetic Neurosteroid (3β,5β,17β)-3-Hydroxyandrostane-17-Carbonitrile. Br. J. Anaesth. 126, 245–255. 10.1016/j.bja.2020.07.022 32859366 PMC7844375

[B57] Timic Stamenic T. Todorovic S. M. (2022). Thalamic T-type Calcium Channels as Targets for Hypnotics and General Anesthetics. Ijms 23, 2349. 10.3390/IJMS23042349 35216466 PMC8876360

[B58] Todorovic S. M. Pathirathna S. Brimelow B. C. Jagodic M. M. Ko S. H. Jiang X. (2004). 5beta-Reduced Neuroactive Steroids Are Novel Voltage-dependent Blockers of T-type Ca2^+^ Channels in Rat Sensory Neurons *In Vitro* and Potent Peripheral Analgesics *In Vivo* . Mol. Pharmacol. 66, 1223–1235. 10.1124/mol.104.002402 15280444

[B59] Tuem K. B. Atey T. M. (2017). Neuroactive Steroids: Receptor Interactions and Responses. Front. Neurol. 8, 442–510. 10.3389/fneur.2017.00442 28894435 PMC5581316

[B60] Vertes R. P. Hoover W. B. Rodriguez J. J. (2012). Projections of the Central Medial Nucleus of the Thalamus in the Rat: Node in Cortical, Striatal and Limbic Forebrain Circuitry. Neuroscience 219, 120–136. 10.1016/j.neuroscience.2012.04.067 22575585

[B61] Weiergräber M. Henry M. Ho M. S. Struck H. Hescheler J. Schneider T. (2008). Altered Thalamocortical Rhythmicity in Ca(v)2.3-deficient Mice. Mol. Cell. Neurosci. 39, 605–618. 10.1016/J.MCN.2008.08.007 18834942

[B62] Weiergräber M. Henry M. Krieger A. Kamp M. Radhakrishnan K. Hescheler J. (2006). Altered Seizure Susceptibility in Mice Lacking the Ca(v)2.3 E-type Ca2^+^ Channel. Epilepsia 47, 839–850. 10.1111/j.1528-1167.2006.00541.x 16686648

[B63] Weiergräber M. Henry M. Radhakrishnan K. Hescheler J. Schneider T. (2007). Hippocampal Seizure Resistance and Reduced Neuronal Excitotoxicity in Mice Lacking the Cav2.3 E/R-type Voltage-Gated Calcium Channel. J. Neurophysiol. 97, 3660–3669. 10.1152/JN.01193.2006 17376845

[B64] Williams M. E. Marubio L. M. Deal C. R. Hans M. Brust P. F. Philipson L. H. (1994). Structure and Functional Characterization of Neuronal Alpha 1E Calcium Channel Subtypes. J. Biol. Chem. 269, 22347–22357. 10.1016/S0021-9258(17)31796-9 8071363

[B65] Wilson S. M. Toth P. T. Oh S. B. Gillard S. E. Volsen S. Ren D. (2000). The Status of Voltage-dependent Calcium Channels in Alpha 1E Knock-Out Mice. J. Neurosci. 20, 8566–8571. 10.1523/jneurosci.20-23-08566.2000 11102459 PMC6773068

[B66] Wu L. G. Borst J. G. Sakmann B. (1998). R-type Ca2^+^ Currents Evoke Transmitter Release at a Rat Central Synapse. Proc. Natl. Acad. Sci. U. S. A. 95, 4720–4725. 10.1073/PNAS.95.8.4720 9539805 PMC22557

[B67] Zaman T. Lee K. Park C. Paydar A. Choi J. H. Cheong E. (2011). CaV2.3 Channels Are Critical for Oscillatory Burst Discharges in the Reticular Thalamus and Absence Epilepsy. Neuron 70, 95–108. 10.1016/J.NEURON.2011.02.042 21482359

[B68] Zamponi G. W. (2016). Targeting Voltage-Gated Calcium Channels in Neurological and Psychiatric Diseases. Nat. Rev. Drug Discov. 15, 19–34. 10.1038/nrd.2015.5 26542451

