# Supplementary material for: Further Evidence that Inhibition of Neuronal Voltage-Gated Calcium Channels Contributes to the Hypnotic Effect of Neurosteroid Analogue, 3β-OH
Source: Front Pharmacol. 2022 May 23;13:850658. doi: 10.3389/fphar.2022.850658 (PMC9169093; doi:10.3389/fphar.2022.850658)
Supplement: Supplementary file 1 [file Image1.pdf]

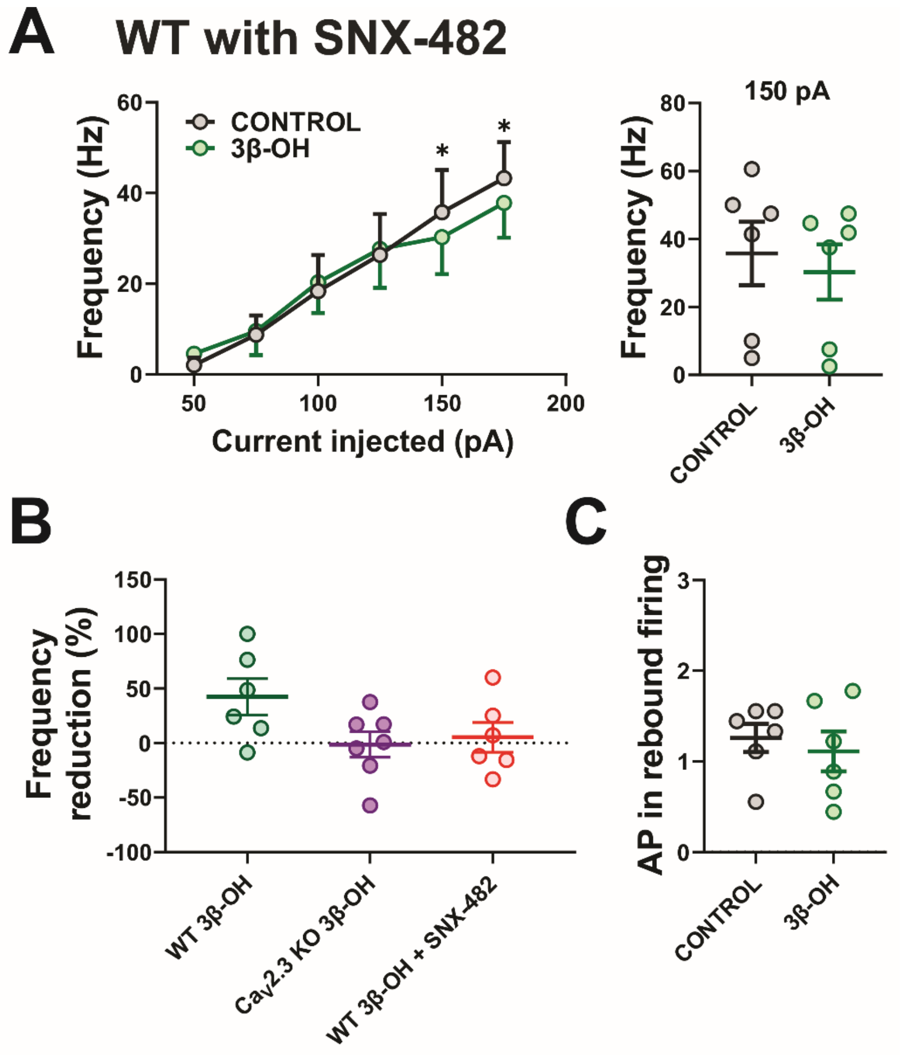

Figure S1. Stimulated tonic and rebound burst firing in WT thalamic slices pretreated with SNX-482 before and during the application of 3β-OH.

(A) Left - average stimulated tonic frequency firing before (black) and during the application of 3 μM of 3β-OH (green) across different current injection (50-175 pA) in thalamic slices from WT animals in the presence of SNX-482 (n=6, two-way RM ANOVA: interaction $F_{5,25}$=14.39, p<0.001; 3β-OH $F_{1,5}$=0.11, p=0.755; current injection $F_{5,25}$=4.09, p=0.007, Šídák's multiple comparisons test presented). (**A**) Right - average tonic frequency at current injection of 150 pA (n=6, paired two-tailed t-test t_5=1.61, p=0.168). (**B**) Average cumulative reduction in stimulated tonic firing normalized to baseline (%) in untreated slices from WT mice, slices from $Ca_V2.3$ KO mice, and slices from WT mice incubated with SNX-482. (**C**) Average number of AP in rebound burst firing before and after the administration of 3β-OH in thalamic slices from WT mice in the presence of SNX-482. *p<0.05.
